# A Fly Enzyme for Motor Control

**DOI:** 10.1371/journal.pbio.0020450

**Published:** 2004-11-30

Long-range communication requires special technology. Although we usually think of nerve cells communicating over distances measured in millimeters, they must also stretch over centimeters and even meters to enable movement and sensation throughout our bodies. In a disorder known as spastic paraplegia, people experience stiffness and loss of function in their legs because the far ends of the longest nerves in the spinal cord degenerate. One form of this disease, autosomal dominant hereditary spastic paraplegia (AD-HSP), is most frequently caused by mutations in a gene that codes for the enzyme Spastin.

Until recently, the function of Spastin has only been inferred by its similarity to another protein, Katanin, which chops up microtubules. The microtubules in neurons, as in all types of cells, provide a network to transport materials from one place in the cell to another. For neurons to communicate with each other and with muscles, neurotransmitters packaged at one end of the cell make the journey through the cell's long, narrow axon on the backs of microtubules. At the other end of the cell, the neurotransmitters reach small cellular projections (boutons) where they are released as a signal to the receiving cell. Since long spinal cord axons are most often affected by AD-HSP, researchers have suggested that transport through axons might be culpable.

Is there a link between Spastin, microtubule severing, and AD-HSP? To address this question, Nina Tang Sherwood and colleagues studied the function of Spastin in the fruitfly Drosophila by identifying the *spastin* gene in this species and manipulating its expression.

They found that indeed Drosophila Spastin in neurons regulates microtubule networks. Overexpressing *spastin* caused collapse of the embryonic central nervous system and also eliminated the microtubule network, as expected based on the related Katanin protein's microtubule-severing activity. But—surprisingly—knocking out *spastin* did not yield the opposite result. *spastin*-null flies had fewer microtubule bundles, particularly at the far ends of the neurons. They also exhibited smaller and more numerous boutons that were unusually clustered together.

On the basis of their results, the authors speculate that Spastin cuts microtubules to a manageable size. Too much Spastin chops the microtubules into useless fragments, but too little Spastin may leave microtubule polymers too large to be efficiently moved into newly forming boutons. With an intermediate amount, microtubule pieces are the right size for transport throughout the neuron.

Without Spastin, normal motor function ceases. In *spastin*-null flies, neurotransmitter release is impaired and flying is impossible. Flies even tend to drag their hind legs. This weakness in the legs is just one compelling parallel to human AD-HSP. The severity of symptoms in people is highly variable, similar to the variability in phenotypes exhibited by Drosophila with intermediate *spastin* gene mutations. The authors caution, however, that they do not prove that the fly phenotypes observed arise through the same mechanisms that cause human AD-HSP. The utility of Drosophila as a model for human AD-HSP has yet to be demonstrated, but the importance of Spastin in regulating neuronal microtubule networks in vivo is no longer in doubt.

